# My-Lupus: A case of anti-transcriptional intermediary factor 1-gamma antibody-positive  dermatomyositis overlapping with discoid lupus erythematosus in an adult patient

**DOI:** 10.1016/j.jdcr.2023.09.002

**Published:** 2023-09-19

**Authors:** Michelle Toker, Tian R. Zhu, Pooja Srivastava, Bijal Amin, Jeanie Lee, Shudan Wang, Benedict Wu

**Affiliations:** a Department of Medicine, Division of Dermatology, Montefiore Medical Center, Albert Einstein College of Medicine, Bronx, New York; b Department of Pathology, Montefiore Medical Center, Albert Einstein College of Medicine, Bronx, New York; c Department of Medicine, Division of Rheumatology, Montefiore Medical Center, Albert Einstein College of Medicine, Bronx, New York

**Keywords:** autoimmune connective tissue disease, dermatomyositis, discoid lupus erythematosus, systemic lupus erythematosus, TIF1-gamma antibody

*To the Editor:*

We appreciate the article by Gutierrez et al,^1^ which highlights dyspigmentation as a prominent finding in patients with transcriptional intermediary factor 1-gamma (TIF1-γ)-associated dermatomyositis (DM). Here, we present a patient with similar dyspigmentation and significant erythema who exhibited clinico-pathologic-serologic features of TIF1-γ-positive DM and discoid lupus erythematosus (DLE). Although the overlap of systemic lupus erythematosus and DM is a well-reported entity, there is scarce literature regarding the overlap of DLE and DM.^2^

A 40-year-old Hispanic woman presented to the rheumatology-dermatology clinic with a 1-month history of a pruritic eruption on her face, scalp, trunk, and proximal upper extremities. A review of systems was negative for fever, myalgia, arthralgia, oral ulcers, dyspnea, and Raynaud phenomenon. The scalp demonstrated scarring alopecia with dyspigmentation and erythema; other scarred areas with dyspigmentation included the conchal bowl and posterior aspect of the neck (Fig 1, *A-C*). Interestingly, she also had the heliotrope eruption, malar erythema crossing the nasolabial folds, striking poikiloderma of the back, shoulders, and lateral aspect of the upper portion of the arms, and prominent proximal nailfold erythema with dilated capillary loops, dropout, and thromboses (Fig 2, *A-C*). Therefore, physical examination demonstrated features of both DLE and DM. Initial 4-mm punch biopsy from the posterior aspect of the neck revealed interface dermatitis with a perivascular and periadnexal lymphohistiocytic infiltrate, consistent with DLE (Fig 1, *D*). However, given the clinical suspicion for DM, a separate 3-mm punch biopsy from the anterior aspect of the shoulder was performed, which demonstrated interface dermatitis with basement membrane thickening, consistent with DM (Fig 2, *D*). Musculoskeletal examination by the rheumatologists (S.W. and J.L.) showed no objective muscle weakness.

**Fig 1:**
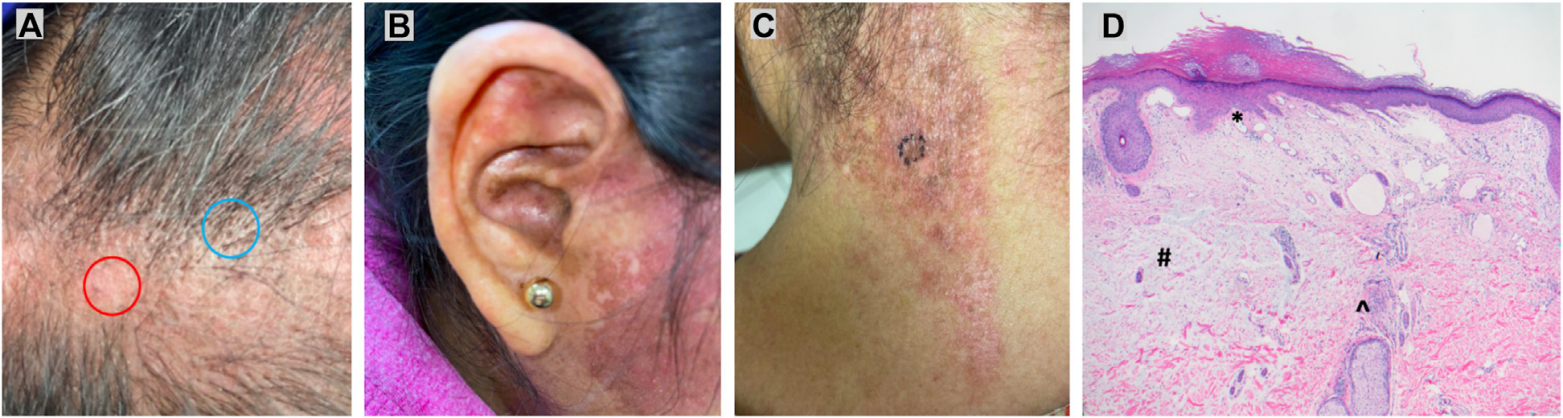
Clinical and histologic images consistent with discoid lupus erythematosus. **A,** Vertex scalp with scarring alopecia, dyspigmentation, and erythema; *blue circle* = follicular plugging; *red circle* = loss of follicular openings. **B,** Conchal bowl with dyspigmentation and scarring. **C,** Posterior aspect of the neck with erythematous and dyspigmented plaques, with *encircled* punch biopsy site. **D,** Punch biopsy of posterior aspect of the neck showing vacuolar alteration at the dermoepidermal junction, *∗*thickened basement membrane, ^*#*^increased dermal mucin, and mild perivascular and ^*ˆ*^periadnexal lymphohistiocytic infiltrate (Hematoxylin-eosin stain; original magnification: ×200).

**Fig 2:**
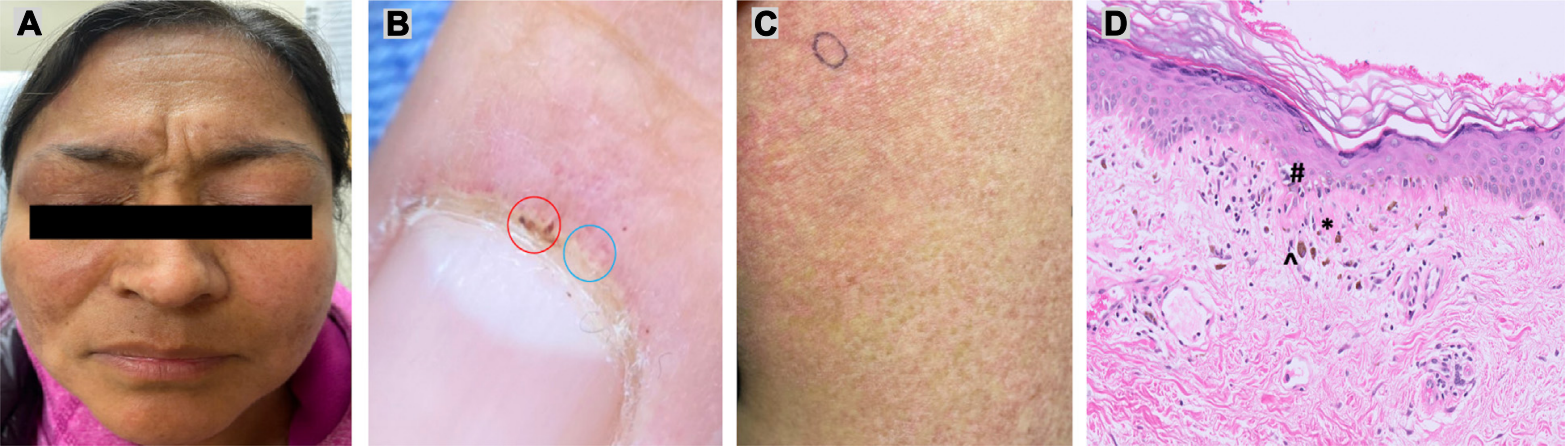
Clinical and histologic images consistent with dermatomyositis. **A,** Eyelids and periorbital skin with violaceous erythema and edema, consistent with heliotrope eruption; malar erythema crossing the nasolabial folds. **B**, Proximal nailfold erythema with dilated capillary loops, capillary dropout (*blue circle*), and thromboses (*red circle*). **C**, Left shoulder with erythematous, poikilodermatous plaques, with *encircled* punch biopsy site. **D**, Punch biopsy of left shoulder showing *∗*thickened basement membrane, ^*#*^vacuolar interface dermatitis, and *ˆ*dermal melanophages (Hematoxylin-eosin stain; original magnification: ×20).

Laboratory analyses were significant for elevated erythrocyte sedimentation rate (49 mm/h), positive antinuclear antibody (titer of 1:640) with a homogeneous pattern, and a negative extractable nuclear antigen antibodies panel. Creatinine kinase, lactate dehydrogenase, and aldolase levels were within normal limits. The MyoMarker Panel 3 was positive for anti–TIF1-γ (74 units). All other myositis-specific (MSA) or associated antibodies were negative. Based on the clinico-pathologic-serologic correlation, she was diagnosed with TIF1-γ antibody-positive amyopathic DM and DLE overlap syndrome. A malignancy workup was unrevealing.

Although DLE overlapping with DM has yet to be documented, a recently published case reported that a patient with systemic lupus erythematosus presented with features of both DLE and DM, including Gottron-like papules; notably, the patient did not undergo MSA testing and received a unifying diagnosis of DLE.^3^ Our case reinforces that cutaneous manifestations of multiple connective tissue diseases may present simultaneously, necessitating several skin biopsies from areas exhibiting different morphologies. A nonscarring truncal and acral eruption with significant poikiloderma and prominent dyspigmentation, pruritus, and erythema may indicate DM, necessitating MSA testing.^1^ Given the associated risk of underlying malignancy, prompt identification of DM is critical. In particular, the TIF1-γ antibody carries the highest known malignancy risk of all MSA.^4^ Therefore, anti-TIF1-γ positivity should prompt the search for underlying malignancy. In conclusion, we present a patient with cutaneous features of both DLE and DM to emphasize that both entities can coexist, which may have significant malignancy screening and management implications.

## Conflicts of interest

None.
